# Supplementary material for: Chromothripsis during telomere crisis is independent of NHEJ, and consistent with a replicative origin
Source: Genome Res. 2019 May;29(5):737–49. doi: 10.1101/gr.240705.118 (PMC6499312; doi:10.1101/gr.240705.118)
Supplement: Supplemental Material [file supp_gr.240705.118_Supplemental_file_1.zip › contigs/annotated_contigs/DB77/contig.2.DB77_length_724_mean_cov_7.66574585635.docx]

**DB77_length_724_mean_cov_7.66574585635**

```
ATGTGATTATGTGAAGAGACTGAATCTATGACTGATTGGTGTACTTGAAAGAGATAGGAAGAATGGAACCAGCTTGGAAAACATATTCC
 >chr11:49183506-49183850 - E=4e-195
AGGATATAATCCATGAGAACTTTCCCAACATAGCTAGATAGGCCAACATTCAAATTCAGGAAATGCAGAGAACCTCAGTAAGATACTAC

TTGAGAAGATCATCCCCAAGACACATACTCATCAGATTCTCCAAGGACAAAATGAGAGAAAAAATGTTAAAGGCAGCTAGAGGGAAAGG

CCAGGTTACCTACAAAGGGAAGCCCATCAGCCTAACAGTGGACCTCTCAGCTGAAACCCTGCAAGCCAGAAGAGAT|T|ATATATATAT
                                                                             >chr11:491
ATATAATCAACAACAAAAGAAAGGGAATCATGACTGTTACACGTACACAGTGTATTATACTGATATGATTTGACTCTGTGTCCCCACCC
77372-49177753 + E=2e-208
AAATCTCACCTTGAATTATGATAATCCCCACGTGTCATGGGAGAGAATTTATGGGAGGTAATTGAATCATGGGGGACGAGTTTTCCTGC

ACTGCTCTAGTGATAGTGAATAAGTCTCATGTGAGCTGATGGTTTTATAAAGGGAAGTTCCCCTGTATATGCTCTCTTGCCTGCTGCCA

GGTAAGATGTAACTTTGCTCCTCACTCACCTTCAGCCATGATTGTGATGCCTCCCCAGCCATATGAAACTGTGAGTCAATTAAACATAT

TTCCCTTTTAAATG
```
